# Highly Efficient Leaf Base Protoplast Isolation and Transient Expression Systems for Orchids and Other Important Monocot Crops

**DOI:** 10.3389/fpls.2021.626015

**Published:** 2021-02-15

**Authors:** Rui Ren, Jie Gao, Dongmei Yin, Kai Li, Chuqiao Lu, Sagheer Ahmad, Yonglu Wei, Jianpeng Jin, Genfa Zhu, Fengxi Yang

**Affiliations:** ^1^Guangdong Key Laboratory of Ornamental Plant Germplasm Innovation and Utilization, Environmental Horticulture Research Institute, Guangdong Academy of Agricultural Sciences, Guangzhou, China; ^2^College of Agronomy, Henan Agricultural University, Zhengzhou, China; ^3^National Key Laboratory for Crop Genetics and Germplasm Enhancement, Nanjing Agricultural University, Nanjing, China

**Keywords:** *CsDELLA*-mediated gibberellin signaling, gene silencing, leaf base, orchids, protoplast isolation, transient expression

## Abstract

Versatile protoplast platforms greatly facilitate the development of modern botany. However, efficient protoplast-based systems are still challenging for numerous horticultural plants and crops. Orchids are globally cultivated ornamental and medicinal monocot plants, but few efficient protoplast isolation and transient expression systems have been developed. In this study, we established a highly efficient orchid protoplast isolation protocol by selecting suitable source materials and optimizing the enzymatic conditions, which required optimal D-mannitol concentrations (0.4–0.6 M) combined with optimal 1.2% cellulose and 0.6% macerozyme, 5 μM of 2-mercaptoethanol and 6 h digestion. Tissue- and organ-specific protoplasts were successfully isolated from young leaves [∼3.22 × 10^6^/g fresh weight (FW)], flower pedicels (∼5.26 × 10^6^/g FW), and young root tips (∼7.66 × 10^5^/g FW) of *Cymbidium* orchids. This protocol recommends the leaf base tissues (the tender part of young leaves attached to the stem) as better source materials. High yielding viable protoplasts were isolated from the leaf base of *Cymbidium* (∼2.50 × 10^7^/g FW), *Phalaenopsis* (1.83 × 10^7^/g FW), *Paphiopedilum* (1.10 × 10^7^/g FW), *Dendrobium* (8.21 × 10^6^/g FW), *Arundina* (3.78 × 10^6^/g FW) orchids, and other economically important monocot crops including maize (*Zea mays*) (3.25 × 10^7^/g FW) and rice (*Oryza sativa*) (4.31 × 10^7^/g FW), which showed marked advantages over previous mesophyll protoplast isolation protocols. Leaf base protoplasts of *Cymbidium* orchids were used for polyethylene glycol (PEG)-mediated transfection, and a transfection efficiency of more than 80% was achieved. This leaf base protoplast system was applied successfully to analyze the *CsDELLA*-mediated gibberellin signaling in *Cymbidium* orchids. We investigated the subcellular localization of the CsDELLA-green fluorescent protein fusion and analyzed the role of *CsDELLA* in the regulation of gibberellin to flowering-related genes via efficient transient overexpression and gene silencing of *CsDELLA* in *Cymbidium* protoplasts. This protoplast isolation and transient expression system is the most efficient based on the documented results to date. It can be widely used for cellular and molecular studies in orchids and other economically important monocot crops, especially for those lacking an efficient genetic transformation system *in vivo*.

## Introduction

Protoplasts are plant cells from which the cell walls have been enzymatically removed (Eeckhaut et al., 2013). They are totipotent, sensitive, and versatile. Protoplast-based platforms allow creation of new plant species via protoplast fusion and regeneration (Melchers et al., 1978; Grosser and Gmitter, 1990). They explore the signal transduction and metabolic pathways transiently respond to hormones and stress factors (Sheen, 2001; Hirata et al., 2012), answer specific questions related to cell types (Petersson et al., 2015; Denyer et al., 2019), and determine the subcellular localization, transport, and interactions of tagged proteins (Goodman et al., 2004; Zhang et al., 2011). The versatility of plant protoplasts greatly facilitates modern botany development (Duarte et al., 2016).

Although a number of efficient protoplast isolation protocols for model plants and many crops are established (Table 1), it is still challenging for numerous horticultural plants and crops. Leaf tissues are the most commonly used source materials, and high yielding mesophyll protoplasts can be easily isolated from tobacco (Nagata and Takebe, 1971), *Arabidopsis* (Menczel, 1980), maize (*Z. mays* L.) (Kanai and Edwards, 1973), rice (*Oryza sativa* L.) (Toriyama and Hinata, 1985), and many other non-model plant species, such as *Medicago sativa* (Song et al., 1990), *Panicum virgatum* (Mazarei et al., 2008), *Elaeis guineensis* (Masani et al., 2014), *Hevea brasiliensis* (Zhang et al., 2016b), *Phaseolus vulgaris* (Nanjareddy et al., 2016), and *Magnolia* (Shen et al., 2017). However, it is not possible to isolate sufficient viable protoplasts from the mature leaves of many other plants due to species limitations. Hence, *in vitro* grown seedlings were tested, and mesophyll protoplasts have been isolated from *Tanacetum* (Keskitalo et al., 2001), grape (*Vitis vinifera*) (Mliki et al., 2003), pepper (*Capsicum annuum* L.) (Jeon et al., 2007), and pineapple (Priyadarshan et al., 2018). However, callus induction is time-consuming (several months), and flower petals have been selected as an alternative for protoplast isolation from ornamental plants, including *Rosa rugosa* (Borochov et al., 1976), *Petunia hybrid* (Oh and Kim, 1994), *Hippeastrum*, and *Tulipa* (Wagner, 1979). However, flowers withered within a short period, and petals are not sufficient to supply continuously for protoplast isolation.

**TABLE 1:** Comparison of past protoplast isolation protocols.

Species	Source material(s)	Enzyme combinations^#^	Maximum yield (/g FW) and viability (%)	References
*Cymbidium*	Leaf (*in vitro*)	1.2/3.0%C + 0.3/1.2%M + 0.5%P	5.2 × 10^4^, –	Pindel, 2007
	Flower pedicel (*in vitro*)		2.7 × 10^6^, –	Pindel, 2007
	Column (*in vitro*)		1.1 × 10^7^, –	Pindel, 2007
	Leaf	0.8/3.2%Cly + 0.3/1.2%M + 0.5%P	4.4 × 10^4^, –	Pindel, 2007
	Root		0.6 × 10^4^, –	Pindel, 2007
	Flower petal	1.20%C + 0.60%M	3.6 × 10^7^, 94.2	Ren et al., 2020
	Flower pedicel		5.3 × 10^6^, 90.3	This study
	Young leaf		3.3 × 10^6^, 91.3	This study
	Leaf base		2.5 × 10^7^, 92.1	This study
	Root		7.8 × 10^5^, 89.3	This study
*Phalaenopsis*	Flower petals	1.00%C + 0.25%M	1.9 × 10^5^, 90.9	Lin et al., 2018b
	Leaf (*in vitro*)	1.00%C + 0.70%M	5.9 × 10^6^, 57.9	Li et al., 2018
	Leaf (*in vitro*)	2.00%C + 1.00%M	1.1 × 10^6^, 83.8	Machmudi et al., 2019
	Leaf base	1.20%C + 0.60%M	1.8 × 10^7^, 92.8	This study
*Dendrobium*	Leaf (*in vitro*)	1.00%C + 0.20%M	4.0 × 10^5^, 95.6	Khentry et al., 2006
	Leaf (*in vitro*)	1.00%C + 0.50%M + 0.10%P	1.6 × 10^4^, –	Aqeel et al., 2016
	Leaf	1.20%C + 0.60%M	8.2 × 10^6^, 91.1	This study
*Maize*	Leaf	0.10%C + 0.01%M	1.0–5.0 × 10^6^, 95.0	Cao et al., 2014
	Leaf	1.20%C + 0.60%M	0.7 × 10^7^, 89.2	This study
	Leaf base	1.20%C + 0.60%M	3.2 × 10^7^, 94.3	This study
*Rice*	Leaf	1.50%C + 0.75%M	1.0 × 10^7^, –	Zhang et al., 2011
	Leaf base	1.20%C + 0.60%M	4.3 × 10^7^, –	This study
	Leaf		No viable protoplast	This study
*Cassava*	Leaf (*in vitro*)	1.60%C + 0.80%M	4.4 × 10^7^, 92.6	Wu et al., 2017
*Pineapple*	Leaf (*in vitro*)	1.50%C + 0.50%M	6.5 × 10^5^, 51.0	Priyadarshan et al., 2018
*Arabidopsis*	Leaf	1.00%C + 0.25%M	3.0 × 10^7^, –	Wu et al., 2009
*Wheat*	Leaf	1.00%C + 0.25%M	7.3 × 10^6^, 95.0	Jia et al., 2016
*Populus*	Leaf (*in vitro*)	2.00%C + 0.50%P	1.0 × 10^8^, >82.0	Tan et al., 2013
	Leaf	3.00%C + 0.80%P	1.0 × 10^7^, >90.0	Guo et al., 2012
*Grapevine*	Leaf (*in vitro*)	1.50%C + 0.40%M	3.3 × 10^6^, 96.0	Zhao et al., 2016
	Cell suspension	2.00%C + 1.00%M	3.0-4.0 × 10^7^, >95.0	Wang et al., 2015
*Chinese kale*	Leaf	2.00%C+0.10%P	6.0 × 10^7^, 95.0	Sun et al., 2018
*Cucumber*	Leaf (*in vitro*)	1.50%C + 0.40%M	6.0-7.0 × 10^6^, 90.0	Huang et al., 2013
*Liriodendron*	Leaf (*in vitro*)	1.50%C + 0.50%M + 0.10%P	1.2 × 10^7^, 97.0	Huo et al., 2017
*Sweet cherry*	Cell suspension	1.00%C + 0.50%P	4.3 × 10^6^, 84.1	Yao et al., 2016
*Medicago*	Legumes root	1.50%C + 2.00%M	1.0 × 10^6^, >90.0	Jia et al., 2018
*Phaseolus vulgaris*	Leaf	1.50%C + 0.37%M	3.0 × 10^5^, –	Nanjareddy et al., 2016
	Flower petals	1.50%C + 0.37%M + 30UP	2.0 × 10^5^, –	
	Hypocotyl and root	2.00%C + 0.30%M + 4.00%H	2.0 × 10^5^, –	
	Nodule	1.00%C + 0.30%M + 4.00%H	1.0 × 10^5^, –	
*Rubber tree*	Leaf	1.50%C + 0.30%M	18.6 × 10^7^, 97%	Zhang et al., 2016b
*Switchgrass*	Cell suspension	6.00%C + 1.00%M + 1.00%D + 0.50%P	8.4 × 10^5^, –	Mazarei et al., 2008
*Pepper*	Leaf (*in vitro*)	1.20%C + 0.30%M	1.5 × 10^6^ – 2.5 × 10^8^,−	Jeon et al., 2007

*^#^C, cellulase R-10; M, macerozyme R-10; P, pectinase; Cly, cellulysin; D, driselase; H, hemicellulose.*

Orchidaceae is one of the largest and the most evolved families of monocot plants on the planet, and more than 70,000 orchid species have been globally cultivated as ornamental and medicinal plants over the past 1500 years (Hsiao et al., 2006; Roberts and Dixon, 2008). *Cymbidium* orchids are important symbols of oriental culture. In the past few decades, extensive research on orchid tissue culture, protoplast fusion, and regeneration has been undertaken (Shrestha et al., 2007; Yam and Arditti, 2009). Nevertheless, few highly efficient protoplast isolation protocols are available for orchids. Orchid protoplasts were first successfully isolated from the leaves of *Cymbidium* (Capesius and Meyer, 1977) and various tissues of *Renantanda*, *Dendrobium*, and *Paphiopedilum* orchids (Teo and Neumann, 1978a, b). Leaf tissues of *in vitro* grown seedlings have been used most commonly (Table 1). Mesophyll protoplasts were successfully isolated from *Dendrobium* [∼3.97 × 10^5^/g fresh weight (FW)] (Khentry et al., 2006), *Cymbidium* (maximum 1.1 × 10^7^/g FW) (Pindel, 2007), and *Phalaenopsis* orchids (5.9 × 10^6^/g FW) (Li et al., 2018). Additionally, flower petals were used for efficient protoplast isolation from *Dendrobium* (Hu et al., 1998) and *Phalaenopsis* [1.9 × 10^5^ per petal (∼0.2 g/FW)] (Lin et al., 2018b), and *Cymbidium* orchids (∼3.50 × 10^7^/g FW) (Ren et al., 2020). However, the protoplast isolation efficiency is relatively lower than that of *Arabidopsis* (∼3.0 × 10^7^/g FW) (Wu et al., 2009), maize (1–5 × 10^6^/g FW) (Cao et al., 2014), rice (∼1.0 × 10^7^/g FW) (Zhang et al., 2011), and cassava (*Manihot esculenta Crantz*) (4.4 × 10^7^/g FW) (Wu et al., 2017). Abundant calcium oxalate crystals accumulated in the ruptured protoplasts can puncture other protoplasts (Pindel, 2007), which is another limiting factor for high yielding protoplast isolation. Enzyme combinations and osmotic conditions influence the yield of viable protoplasts (Nagata and Takebe, 1971; Kanai and Edwards, 1973; Sheen, 2001; Chen et al., 2006; Yoo et al., 2007).

The protoplast-based transient expression system (PTES) provides an ideal platform for molecular, cellular, and functional identification of genes/proteins. It relies on the transient expression of exogenous genetic information via the introduction of nucleic acids vectors by polyethylene glycol (PEG) or electroporation (Krens et al., 1982; Hauptmann et al., 1987; Hayashimoto et al., 1990). Compared with stable transformation, PTES is fast, convenient and economical. High throughput PTES has been applied to screen transactivation of hundreds of transcription factors (Wehner et al., 2011), and highly efficient genome editing and gene silencing cassettes prior to the development of transgenic plants (Bart et al., 2006; Tang et al., 2016; Cao et al., 2014; Lin et al., 2018b; Page et al., 2019). Lin et al. (2018a) reported an efficient PTES to enable molecular, cellular, and functional studies for *Phalaenopsis* orchids. However, a versatile PTES has not been established for *Cymbidium* orchids.

**FIGURE 1:**
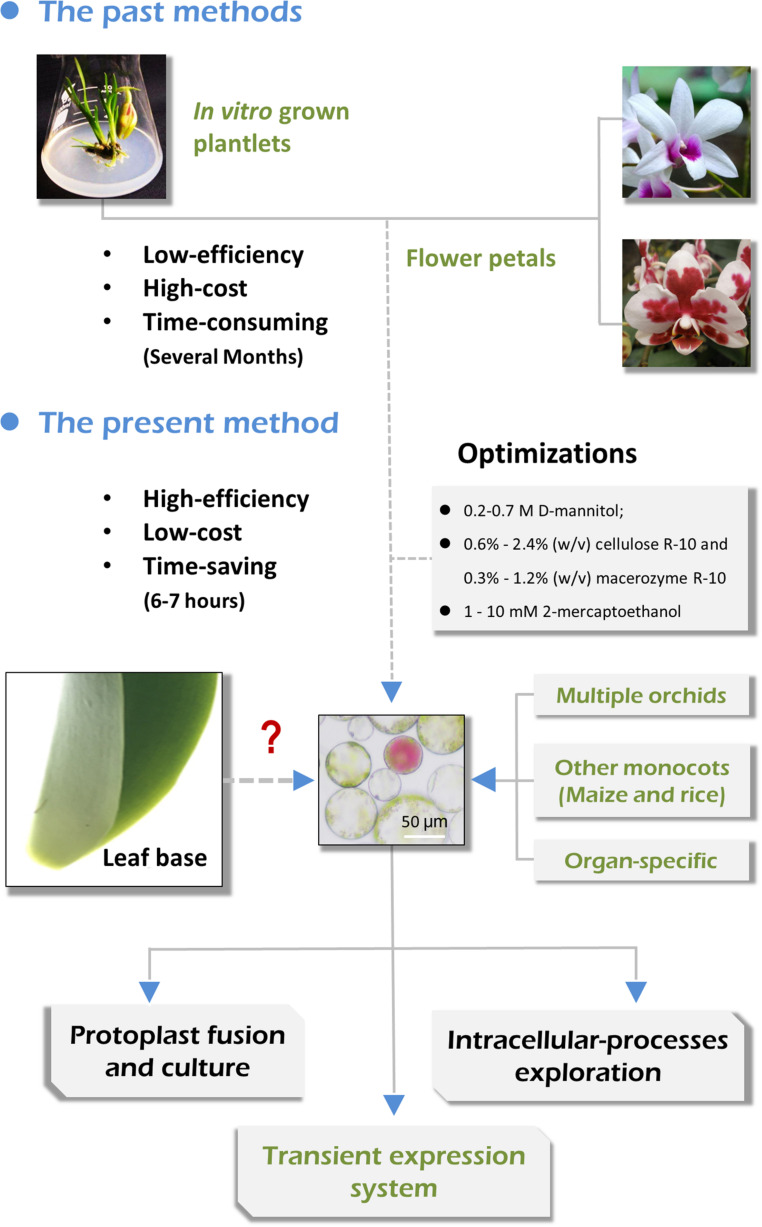
Advantages of the present orchid protoplast isolation method compared with past methods.

In this study, we aimed to develop a simple and efficient protoplast isolation and transient expression system for Orchids. By selecting suitable source materials (including flower pedicels, young leaves, leaf base tissues, and young root tips) and optimizing the enzymatic conditions, high yielding protoplasts were successfully isolated from *Cymbidium* plants (Figure 1). This protocol recommends the leaf base tissues as suitable source materials that provide the highest yield of viable protoplasts, which is readily available, sustainable, and inexpensive. We obtained sufficient tissue- and organ-specific protoplasts form various tissues, including flower pedicels, young leaves, tender leaf bases, and young root tips, by determining the optimal enzymatic conditions. Additionally, this protocol has been applied to highly efficient leaf base protoplast isolation from *Phalaenopsis*, *Paphiopedilum*, *Dendrobium*, and *Arundina* orchids, and important monocot crops like maize and rice. Finally, the *Cymbidium* leaf base protoplasts were used to establish the PEG-mediated PTES, which is feasible for investigating protein subcellular localization, cellular signal transduction in response to phytohormones, and efficient gene transient overexpression and silencing. This versatile leaf base protoplast system would be useful for biological studies on orchids and many other monocot plant species.

## Materials and Methods

### Plant Materials and Growth Conditions

Five orchid species (*Cymbidium*, *Phalaenopsis*, *Paphiopedilum*, *Dendrobium*, and *Arundina*) were selected for this study. These orchids exhibited distinct morphological characteristics; for instance, the leaves of *Phalaenopsis* orchids were fleshy, whereas those of *Cymbidium* orchids were hard leather-like, and the leaves of *Dendrobium* and *Paphiopedilum* orchids were in between these. These orchid plants were obtained from the orchid breeding base of the Environmental Horticulture Research Institute, Guangdong Academy of Agricultural Sciences, China. They were grown in plastic pots (20 cm × 20 cm) in greenhouses under the exact growing conditions necessary for each species, as previously described (Yang et al., 2019). Maize and rice were also included, with maize and rice seeds obtained from the Rice Research Institute and Crops Research Institute of Guangdong Academy of Agricultural Sciences, respectively. The maize and rice seedlings were grown in plant growth rooms at 27°C under a 16 h light/8 h dark cycle and 60% humidity.

### Protoplast Isolation

Protoplast isolation from the various plant species was conducted based on protocols established by Yoo et al. (2007) and Lin et al. (2018b) with some modifications. Various source materials collected from *Cymbidium* orchids, including flower pedicels, young leaves, leaf base tissues, and young root tips were tested for tissue- and organ-specific protoplast isolation. Additionally, leaf base tissues of *Phalaenopsis*, *Paphiopedilum*, *Dendrobium*, and *Arundina* orchids, and rice and maize plants were tested for protoplast isolation. Maize and rice plants were grown for 7 days after germination, and then the leaf bases of these seedlings were collected for protoplast isolation, whereas their young leaves served as controls.

The tissues were surface sterilized for 3 min in 75% ethyl alcohol (volume percentage, v/v), followed by five washes in sterilized water. The sterilized tissues were cut into 0.5–1.0 mm strips or slices using fresh surgical blades on sterile filter paper and were immediately transferred to a freshly prepared enzyme solution in a 100 mL sterile flask. The enzyme solution was prepared as follows: 20 mM KCl (Sigma, St. Louis, MO, United States), 20 mM 2-(N-morpholino)ethanesulfonic acid (pH 5.7) (Sigma) with different concentrations of D-mannitol (0.2, 0.3, 0.4, 0.5, 0.6, and 0.7 M) (Sigma), cellulose R-10 (0.6, 1.2 and 2.4, w/v), and macerozyme R-10 (0.3, 0.6, and 1.2%, w/v) (Yakult Pharmaceutical Industry Ltd., Nishinomiya, Japan). The solution was then warmed up to 55°C for 10 min, and cooled to room temperature (∼25°C). Then, 10 mM CaCl_2_ (Sigma), and 0.1% (w/v) bovine serum albumin (Sigma) were added to different concentrations of 2-mercaptoethanol (0, 1, 2, 5, and 10 mM) (Sigma). For each treatment, 10 mL enzyme solution was prepared for approximately 0.5 g fresh tissues. After incubation at 28°C in the dark with a rotation of 30 rpm for different periods (2, 4, 6, and 8 h), the released protoplasts were harvested. Generally, the enzyme mixture containing protoplasts was diluted with an equal volume of wash solution (W5) that contained 154 mM NaCl (Sigma), 125 mM CaCl_2_, 5 mM KCl, and 4 mM MES (pH = 5.7). The protoplast-containing solution was filtered through a 150 μm nylon mesh into a 50 mL round-bottomed centrifuge tube and centrifuged at 100 *× g* for 2 min to pellet the protoplasts (at room temperature ∼25°C). Subsequently, the supernatant was removed carefully as much as possible with a sterile syringe. The protoplast pellet was re-suspended in 20 mL of W5 solution (Negrutiu et al., 1986), and then the yield and viability of protoplasts were estimated.

### Protoplast Yield and Viability Estimation

The protoplasts were counted and photographed using a Leica DM2500 microscope (Leica, Wetzlar, Germany) with a hemocytometer to calculate the estimated yield of protoplasts. The protoplast yield was calculated as the total number of protoplasts released in the enzyme mixture divided by the FW of tissues used for protoplast isolation (protoplasts/g FW). Fluorescein diacetate (FDA) (Sigma) staining was applied to determine the viability of protoplasts as follows: 9 μL of the protoplast-containing solution was placed onto a glass slide, followed by the addition of 1 μL of 0.2% FDA solution [dissolved in acetone (Solarbio Science and Technology Co., Ltd. Beijing, China)] and incubated at room temperature for 1–2 min. Viable protoplasts with green fluorescence were visualized and photographed using an LSM 710 confocal laser microscope (Carl Zeiss, Inc., Jena, Germany) with blue excitation block. Protoplast viability was measured as the number of protoplasts with green fluorescence divided by the total number of protoplasts ×100%. Three images were selected for the yield and viability measurements of each sample. Each experiment was repeated three times.

### Plasmid Preparation

To test the feasibility of orchid leaf base protoplasts for protein subcellular localization and gene regulation analysis, vector plasmids expressing green fluorescent protein (GFP), GFP-protein fusions, proteins, and gene-specific target sequence were prepared for PEG-mediated protoplast transfection (Supplementary Figure 1A). The empty vector pAN580-GFP expressing GFP was used as a control for the protein subcellular localization. Plant organelle markers, including pGreenII62-SK-AtWAK2-GFP and pGreenII62-SK-AtPIP2A-GFP, were used in the present study. The two AtWAK2 and AtPIP2A signal peptides isolated from *Arabidopsis thaliana* were specifically accumulated in the endoplasmic reticulum and plasma membrane, respectively (Gomord et al., 1997; Cutler et al., 2000).

The recombinant plasmid pAN580-*CsDELLA*-GFP was obtained by inserting the full-length coding sequence (CDS) of *CsDELLA* (GenBank accession number: MK282635.1) without a termination codon between the dual *cauliflower mosaic virus* 35S promoter and the GFP gene of the vector pAN580-GFP. Meanwhile, the full-length CDS of *CsDELLA* was cloned into vector pAN580-GFP, and replaced with the GFP gene, resulting in the pAN580-*CsDELLA* plasmid (Supplementary Figure 1B). For the gene silencing vector pTCK303-*CsDELLA*-RNAi, the 243 base pair sequence of *CsDELLA* was cloned into the vector pTCK303-RNAi with an *Ubiquitin* promoter (Supplementary Figure 1C). Briefly, total RNA was extracted from freshly collected young leaves of *Cymbidium sinense* using an RNA Simple Total RNA Kit (Tiangen, Beijing, China). The DNA-free RNA was used for first-strand cDNA synthesis with oligo (dT) primers and a PrimeScript^TM^ 1st strand cDNA Synthesis Kit (Takara, Dalian, China), following the manufacturer’s instructions. Specific primers with overlapping homologous ends were designed using the Primer Premier 5.0 software (Premier, Palo Alto, CA, United States) according to the full-length CDS and gene-specific target sequence of *CsDELLA* (Supplementary Table 1). Subsequently, the fragment of *CsDELLA* was amplified using PrimerSTAR Max DNA Polymerase (Takara) with the cDNA and specific primers. The polymerase chain reaction (PCR) products were purified and cloned into certain vectors using a Seamless Assembly Cloning Kit (CloneSmarter, Houston, TX, United States) following the manufacturer’s instructions.

Vectors including pGreenII62-SK-AtWAK2-GFP, pGreenII62-SK-AtPIP2A-GFP, pAN580-GFP, pAN580-CsDELLA-GFP, pAN580-*CsDELLA*, and pTCK303-*CsDELLA*-RNAi were transformed into *Escherichia coli* DH5α competent cells (Tiangen) according to the manufacturer’s instructions, respectively. Each single *E. coli* colony was picked and inoculated in 50 mL Luria-Bertani broth [Tryptone (1.0%, w/v), Yeast extract (0.5%, w/v), and NaCl (1.0%, w/v), pH = 7.0] containing the appropriate antibiotic. Bacterial cells were pelleted by centrifuging cultures at 3,000 × *g* for 10 min following culturing at 37°C and 200 rpm shaking for 12–16 h. Following mass replication of the bacterium, plasmid DNA was extracted using an Endo-Free Plasmid Maxi Kit (Omega Bio-tek, Norcross, GA, United States). The concentrated plasmid DNA was prepared at different concentrations (up to 2.0 μg/μL), and was transformed into protoplasts.

### PEG-Mediated Protoplast Transfection

Following yield and viability measurements, protoplasts were further purified before transfection and transient expression. The 20 mL protoplast-containing W5 solution was re-centrifuged at 100 *× g* for 2 min (at room temperature). Following resuspension in 20 mL W5 solution, protoplasts were placed on ice for 30 min. Viable protoplasts settled at the bottom of the tube by gravity. Finally, the supernatant was carefully removed as much as possible, and the purified protoplasts were adjusted to a density of 1 × 10^5^ – 1 × 10^6^/mL with the pre-chilled MMG solution (Negrutiu et al., 1987). The MMG solution comprised 0.5 M D-mannitol, 15 mM MgCl_2_, and 4 mM MES (pH = 5.7).

Protoplast transfection was conducted following a modified PEG-mediated protocol (Yoo et al., 2007). For each transfection, 100 μL of MMG-protoplast mixture was gently mixed with 10 μL of pre-chilled plasmid DNA in 2 mL round-bottomed centrifuge tubes. Then, an equal volume (110 μL) of freshly prepared PEG-CaCl_2_ solution was added and immediately mixed by gentle inversion. The PEG-CaCl_2_ solution was composed of 100 mM CaCl_2_, 0.2 M D-mannitol, and 25% PEG4000 (final concentration, w/v) (Sigma). The mixture was incubated at room temperature in the dark for 10 min. The transfection was stopped by adding two volumes of W5 solution (440 μL) followed by centrifugation at 100 *× g* for 2 min. The transfected protoplasts were then washed with W5 solution and re-suspended in 1 mL WI solution for each of the 6-well tissue culture plates. The WI solution was composed of 0.4 M D-mannitol, 20 mM KCl, and 4 mM MES (pH = 5.7). For transient expression of genes/proteins, the protoplast mixture was incubated in the dark at 23°C for 12–36 h.

### Confocal Imaging of Transfected Protoplasts

After incubation in the dark at 23°C for 12–24 h, the fluorescence of GFP or GFP-protein fusions was viewed under an LSM710 confocal laser scanning microscope (Carl Zeiss, Inc.). Transformation efficiency of the protoplasts was determined based on the GFP-reporter expression using the transient expression vector pAN580-GFP. GFP fluorescence was observed and 3–5 images were taken randomly under an LSM780 fluorescent microscope (Carl Zeiss, Inc.) or LSM710 confocal laser scanning microscope. The transformation efficiency was measured as a bright green fluorescent protoplast number in view/total protoplast number in view (%). At least three photographs were taken for each sample, and these experiments were independently conducted at least three times. For subcellular localization analysis, plant organelle markers pGreenII62-SK-AtWAK2-GFP and pGreenII62-SK-AtPIP2A-GFP, and the control vector pGreenII62-SK-GFP were transfected into orchid leaf base protoplasts. Red chlorophyll fluorescence was used to indicate the intercellular location of chloroplasts.

### Protoplast Treatment

Protoplast treatments were undertaken in W5 solution supplemented with different concentrations of gibberellin (GA3) (1, 10, and 100 μM). Approximately 10^7^ protoplasts in 1 mL W5 solution were used for each treatment. W5 solution supplemented with water was used as the control treatment at each time point. Protoplasts were cultivated in 6-well plates in a growth chamber at 23°C in the dark for 6–24 h.

### Quantitative Real-Time Polymerase Chain Reaction

Protoplasts treated with GA3 were harvested at 6, 12 and 24 hours post treatment (hpt), and transfected protoplasts were harvested at 12, 24 and 36 hours post transfection (hpt). First-strand cDNA was obtained from the total RNA of the harvested protoplasts. Gene-specific primers were designed according to the CDSs of *CsDELLA*, *SUPPRESSOR OF OVEREXPRESSION OF CONSTANS1* (*CsSOC1*, MF474250.1), *FLOWERING LOCUS T* (*CsFT*, HM803115.1), *SHORT VEGETATIVE PHASE3* (*CsSVP3*, MF462098.1), *CsLFY* (KC138806.1), and APETALA1 (*CsAP1*, JQ326260.1) using Primer Premier 5.0 software (Premier, Palo Alto, CA, United States). *CsUbiquitin* in *Cymbidium* (referred to as *CsUBQ*, AY907703) was used as an internal reference control to normalize the total amount of cDNA in each reaction (Supplementary Table 1).

The quantitative real-time polymerase chain reaction (qRT-PCR) was performed in a 20 μL reaction volume comprising 2.0 μL of 5× diluted first-strand cDNA (approximately 20 ng), 0.8 μL of each primer (10.0 μM), 10.0 μL of 2× SYBR Green I Master Mix (Takara), and 6.4 μL of sterile distilled H_2_O. All reactions were performed in 96-well reaction plates using a Bio-Rad CFX-96 Real-time PCR System (Bio-Rad, Hercules, Canada) with three technical replicates. The following PCR conditions were used: 95°C for 5 min, followed by 40 cycles at 95°C for 15 s, 60°C for 30 s, and 72°C for 30 s, and then at 68°C for 5 min. The expression of candidate genes was quantified using the relative quantification (2^–ΔΔ*CT*^) method. Each sample was collected independently with three biological replicates.

### Statistical Analysis

Statistical analysis was performed using one-way analysis of variance with SPSS version 18.0 software (SPSS Inc., Chicago, IL, United States). Data are presented as the mean ± standard error from three independent experiments. The least significant differences among treatments were determined at the 5% level of probability with Duncan’s multiple range tests.

## Results

### Selection of Source Materials for *Cymbidium* Protoplast Isolation

**FIGURE 2:**
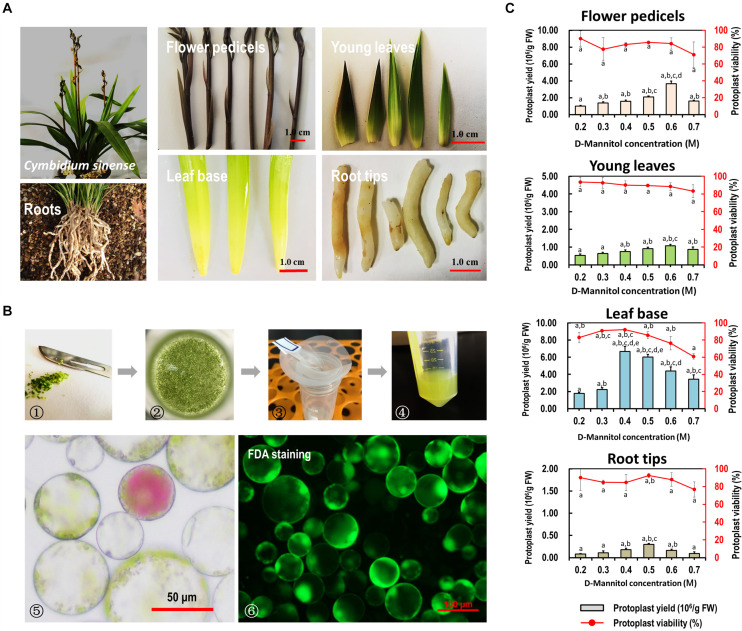
Protoplast isolation from various organs/tissues of *Cymbidium* orchids. **(A)** Various tissues including flower petals, flower pedicels, young leaves, leaf base tissues, and root tips collected from *Cymbidium* orchids were used for protoplast isolation. **(B)** Schematic illustration of protoplast isolation from *Cymbidium* orchid: (1) cut tissues into 0.5–1.0-mm strips or slices using fresh surgical blades on sterile filter papers; (2) transfer strips or slices quickly and gently into in a 100 mL flask; (3) enzymatic solution containing protoplasts were filter through a 150 μm nylon mesh into a 50 mL round-bottomed tube; (4) wash protoplasts twice with W5 solution; (5) check the morphology and yield of protoplasts under a microscope; (6) measure the protoplast viability using the FDA staining method. **(C)** Optimal D-mannitol concentrations in enzyme solutions were determined for different tissues. Data presented as means of three biological replicates with error bars indicating standard deviations (SD), and different letters (a–e) among treatments indicate statically significant differences at *p* < 0.05 based on Duncan’s multiple range test.

To establish an efficient protoplast isolation protocol for orchids, various source materials, including flower pedicels, young leaves, leaf base tissues, and young root tips of *Cymbidium* orchids were tested (Figure 2A). Viable protoplasts were successfully isolated from various tissues or organs of *Cymbidium* plants following the protocol described by Yoo et al. (2007) (Figure 2B). However, the yield and viability of released protoplasts were relatively low, and abundant calcium oxalate crystal raphides were observed in the protoplast-containing solution (Supplementary Figure 2).

To reduce the protoplast rupture or collapse during enzymatic digestion, gradient concentrations of D-mannitol in the enzyme solution (0.2, 0.3, 0.4, 0.5, 0.6, and 0.7 M) were included for different tissues. We found that the D-mannitol concentration significantly affected the efficiency of protoplast isolation. Generally, increasing D-mannitol concentrations resulted in increased protoplast yields, whereas exceeding osmotic pressures resulted in decreased yields. The optimal D-mannitol concentrations for various tissues gave the highest yield and viability simultaneously. For flower pedicels, leaf base, young leaves, and root tips of *Cymbidium* orchids, the optimal D-mannitol concentrations were determined as 0.6, 0.6, 0.4, and 0.5 M, respectively. With the optimal D-mannitol concentrations, the orchid leaf base gave the highest yield of viable protoplasts (∼6.67 × 10^6^/g FW), followed by the flower pedicels (∼3.67 × 10^6^/g FW), young leaves (∼1.07 × 10^6^/g FW), and root tips (∼2.97 × 10^5^/g FW) (Figure 2C). In addition, protoplasts isolated from young leaves and root tips remained intact for more than 24 h in the W5 solution (Supplementary Figure 2), whereas that of flower pedicels ruptured within 6 h post-isolation. Most protoplasts isolated from the leaf base tissues (>70%) remained intact for up to 3 days in the WI solution (Nagy and Maliga, 1976), which was stronger and longer-term healthy than that of the other tissues/organs. Therefore, the leaf base was a better source material for protoplast isolation from *Cymbidium* orchids.

### Optimization of Enzymatic Conditions for *Cymbidium* Protoplast Isolation

**FIGURE 3:**
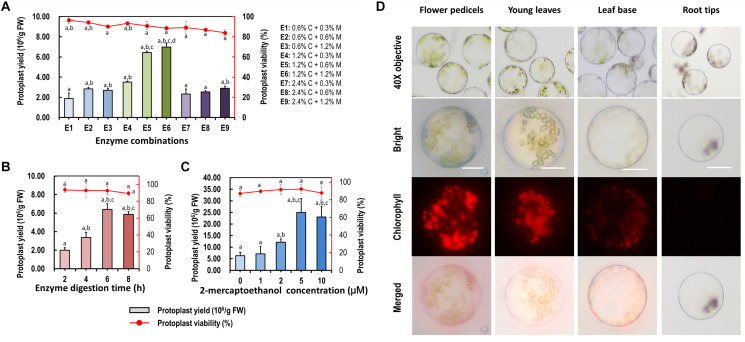
Optimization of *Cymbidium* protoplast isolation conditions. Factors including **(A)** enzyme combination, **(B)** enzyme digestion time, and **(C)** 2-mercaptoethanol concentration were investigated to optimize the yield and viability of protoplasts isolated from the *Cymbidium* leaf base; data presented as means of three biological replicates with error bars indicating standard deviations (SD), and different letters (a–e) among treatments indicate statically significant differences at *p* < 0.05 based on Duncan’s multiple range test. **(D)** Protoplasts were isolated from *Cymbidium* flower pedicels, young leaves, leaf base, and root tips. Protoplasts were photographed using an LSM 710 confocal laser microscope with green excitation block; Bar, 20 μm.

More factors affecting protoplast isolation were investigated to increase the yield and viability of protoplasts isolated from the leaf base of *Cymbidium* orchids. An increase in total enzyme concentrations increased the protoplast yield, whereas excess enzymes resulted in a decrease in protoplast yield and viability. The optimal combination of enzymes was 1.2% (weight/volume, w/v) of cellulose and 0.6% (w/v) of macerozyme, which gave the highest yield (6.97 × 10^6^/g FW) and viability (∼88.49%) of leaf base protoplasts (Figure 3A). Moreover, insufficient digestion time led to incomplete release of protoplasts, whereas excess incubation resulted in the rupture of protoplasts. With optimal D-mannitol concentration and enzyme combination, the highest protoplast yield (6.40 × 10^6^/g FW) and viability (∼92.76%) were obtained after 6 h of enzymatic digestion (Figure 3B). Further optimization revealed that the addition of 2-mercaptoethanol contributed to decreased calcium oxalate raphides and increased yield and viability of protoplasts. Given the combination of optimal D-mannitol concentration (0.4–0.6 M), enzyme mixture (1.2% cellulose and 0.6% macerozyme), digestion time (6 h), and 2-mercaptoethanol (5 μM), the highest *Cymbidium* protoplast yield (∼2.50 × 10^7^/g FW) and viability (∼92.09%) were achieved (Figure 3C).

With this protocol, higher yields of viable protoplasts were isolated form young leaves (∼3.22 × 10^6^/g FW), flower pedicels (∼5.26 × 10^6^/g FW), and young root tips (∼7.66 × 10^5^/g FW) (Figure 3D). The isolated protoplasts ranged from 20 to 100 μm in diameter, and a large proportion of protoplasts isolated from the young leaves and flower pedicels were rich in cytoplasm and anthocyanidin. Protoplasts isolated from the leaf base and root tips were almost translucent with few chloroplast auto-fluorescent signals. Therefore, our protoplast isolation protocol is suitable for investigating intracellular processes in tissue- and organ-specific protoplasts of *Cymbidium* orchids.

### Suitability of the Leaf Base Protoplast Isolation Protocol for Other Orchids and Monocot Crops

**FIGURE 4:**
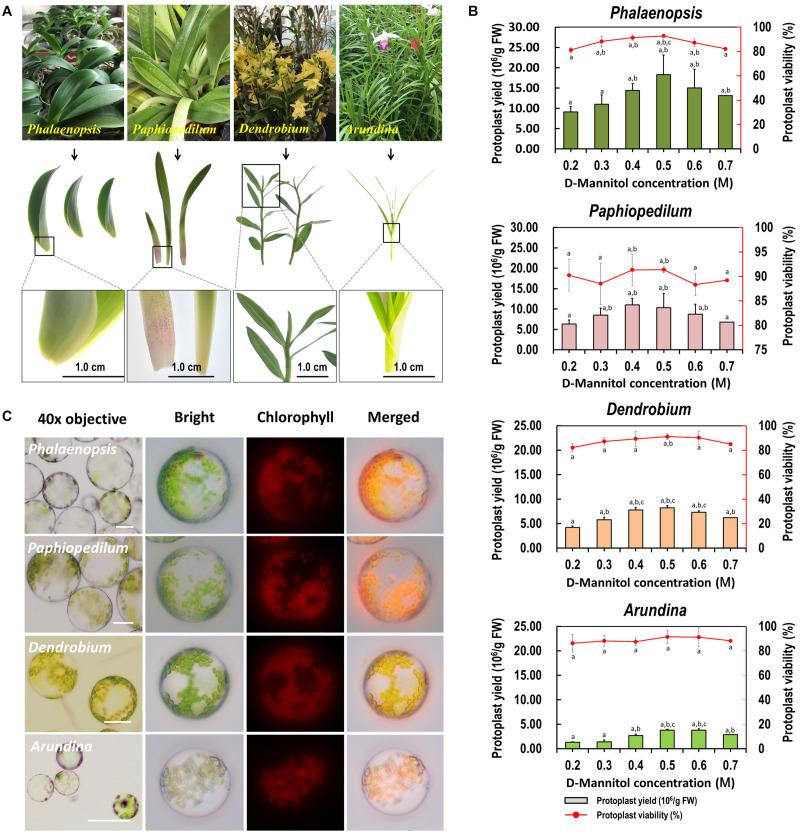
Suitability of the optimized protoplast isolation protocol for other orchid species. **(A)** The leaf base of *Phalaenopsis* and *Paphiopedilum*, and young leaves of *Dendrobium* and *Arundina* were collected for orchid protoplast isolation. **(B)** Different D-mannitol concentrations in the enzyme solutions (0.2, 0.3, 0.4, 0.5, 0.6, and 0.7 M) were established for different orchid species, data presented as means of three biological replicates with error bars indicating the SD values, and different letters (a–e) among treatments indicate statically significant differences at *p* < 0.05 based on Duncan’s multiple range test. **(C)** Protoplasts were isolated from the leaf base of *Phalaenopsis* and *Paphiopedilum*, and from the young leaves of *Dendrobium* and *Arundina* orchids. Protoplasts were photographed using an LSM 710 confocal laser microscope with green excitation block. Bar, 20 μm.

To test the suitability of our protoplast isolation protocol for other species, leaf base tissues collected from *Phalaenopsis*, *Paphiopedilum*, *Dendrobium*, and *Arundina* orchids were tested with different D-mannitol concentrations (0.2–0.7 M) in enzyme solution (Figure 4A). High yielding protoplasts was obtained from these orchids, and optimal D-mannitol concentrations were determined to be 0.4–0.6 M (Figure 4B). The maximum yields of viable protoplasts were isolated from *Phalaenopsis* (1.83 × 10^7^/g FW) and *Paphiopedilum* (1.10 × 10^7^/g FW), which were higher than those of *Dendrobium* (8.21 × 10^6^/g FW) and *Arundina* (3.78 × 10^6^/g FW) (Figure 4B). Protoplasts isolated from *Phalaenopsis* and *Paphiopedilum* (ranged from 40 to 100 μm in diameter) were larger than those isolated from *Dendrobium* and *Arundina* (approximately 20–60 μm in diameter) (Figure 4C). Thus, our protocol exhibits broad suitability in efficient protoplast isolation for orchids exhibiting distinct morphological characteristics.

**FIGURE 5:**
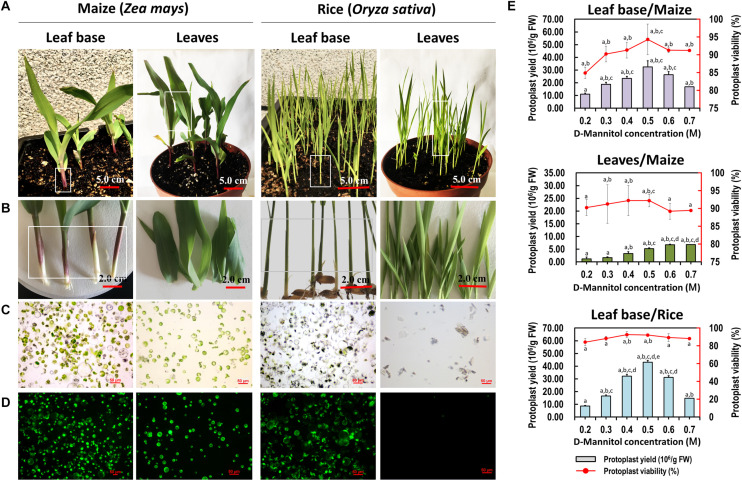
Protoplasts isolated from the leaf base and young leaves of maize and rice. **(A)** Ten-days-old maize and rice seedlings were used for protoplast isolation. **(B)** The leaf base and young leaves of maize and rice were collected for protoplast isolation, respectively. **(C)** Protoplasts were successfully isolated from the leaf base of maize and rice, which was better than that of isolated from the young leaves. **(D)** Viability test of protoplasts isolated from maize and rice using FDA staining. **(E)** Different D-mannitol concentrations in the enzyme solutions (0.2, 0.3, 0.4, 0.5, 0.6, and 0.7 M) were established for protoplast isolation. Data presented as means of three biological replicates with error bars indicating the SD values, and different letters (a–e) among treatments indicate statically significant differences at *p* < 0.05 based on Duncan’s multiple range test.

Sharing similar anatomical characteristics with orchids, economically important monocot crops maize and rice were also tested for the suitability of our protoplast isolation protocol. Leaf base tissues and young leaves were all used, and leaf base protoplast isolation method showed marked advantages over previous mesophyll protoplast isolation protocols (Table 1). High yielding viable protoplasts were released from the leaf base of maize (3.25 × 10^7^/g FW) and rice (4.31 × 10^7^/g FW), and their optimal D-mannitol concentrations were both determined to be 0.5 M (Figure 5). The leaf base protoplasts isolated from maize (ranging from 20 to 60 μm in diameter) were smaller than those of rice (20–90 μm) (Supplementary Figure 3). The majority of rice protoplasts were transparent and highly viable. The efficiency of protoplast isolation from the leaf base tissues was higher than that of the leaves (Figure 5 and Table 1). It indicated that our leaf base protoplast isolation protocol enables highly efficient protoplast isolation from many other monocot plants.

### PEG-Mediated Transient Expression and Protein Subcellular Localization

**FIGURE 6:**
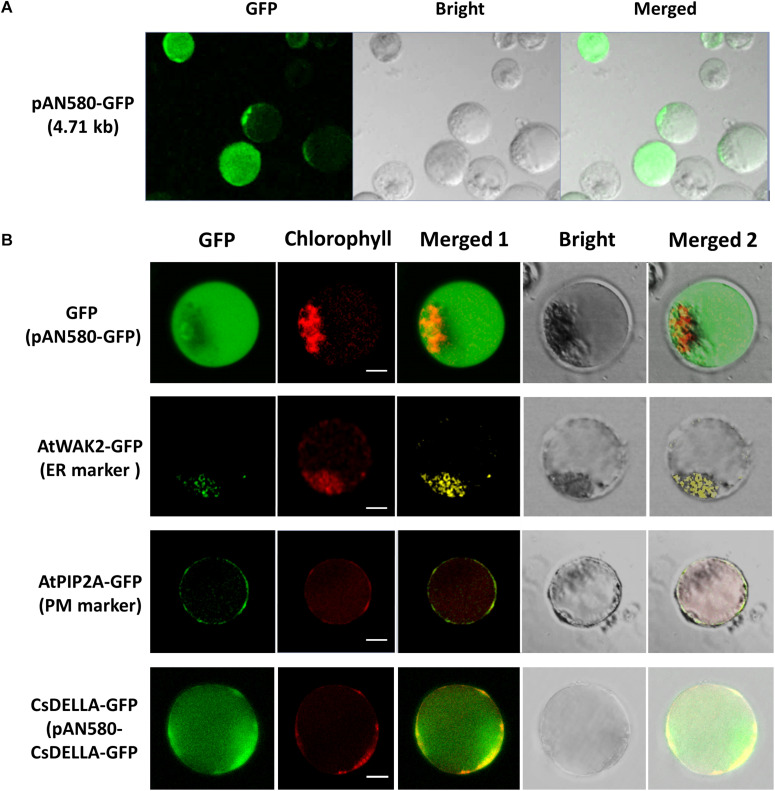
Transient expression and protein subcellular localization studies using *Cymbidium* leaf base protoplasts. **(A)** Transient expression of GFP in the *Cymbidium* leaf base protoplasts transfected with empty control vector pAN580-GFP (expressing GFP). **(B)** Empty control vector, endoplasmic reticulum marker AtWAK2-GFP, plasma membrane marker AtPIP2A-GFP, and CsDELLA-GFP vectors were transformed into *Cymbidium* protoplasts to test the feasibility of the PTES for protein subcellular localization. Bar, 10 μm.

To test the feasibility of the leaf base protoplasts for gene transient expression, PEG-mediated *Cymbidium* protoplast transfection was performed using the vector pAN580-GFP. According to the GFP expression in the transfected protoplasts, a maximum transfection efficiency of more than 80% was obtained by optimizing factors affecting PEG-mediated transfection, including incubation time, final PEG4000 concentration, and amount of plasmid DNA (Figure 6A). In addition, most of the transfected protoplasts maintained integrity up to 36 hpt. These results indicate that leaf base protoplasts of orchids are suitable for transient gene/protein expression.

The efficient transient expression system established in the present study was tested further for the feasibility of the subcellular localization of proteins. Empty control vector pAN580-GFP, the two plant intercellular organelle markers (pGreenII62-SK-*AtWAK2*-GFP and pGreenII62-SK-*AtPIP2A*-GFP), and the recombined vector pAN580-*CsDELLA*-GFP were transfected into *Cymbidium* leaf base protoplasts, respectively. A total of 12–16 h later, green fluorescence was observed in the intracellular compartments of the transfected protoplasts. Fusion proteins AtWAK2-GFP and AtPIP2A-GFP were detected specifically in the endoplasmic reticulum and plasma membrane, respectively, whereas the fusion protein CsDELLA-GFP and vector control were distributed throughout the entire cell (Figure 6B). Thus, the leaf base protoplasts are suitable for molecular and cellular characterization of genes/proteins.

### The Protoplast System Enables Cellular and Molecular Investigations in Cymbidium Orchids

**FIGURE 7:**
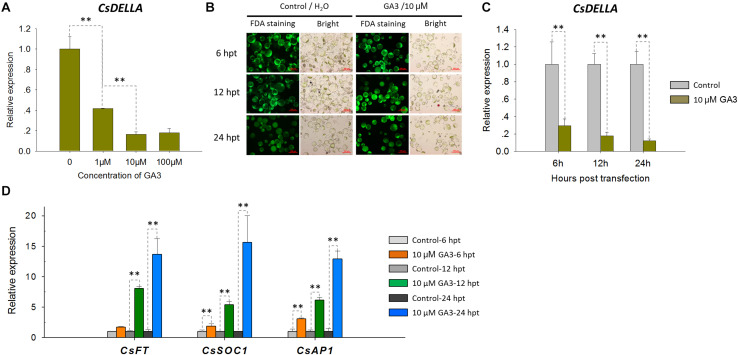
Expression analysis of *CsDELLA*, *CsFT*, *CsSOC1*, and *CsAP1* genes in response to GA3 induction with qRT-PCR. **(A)** Relative expression levels of the *CsDELLA* gene induced by different concentrations of GA3 were measured with qRT-PCR. **(B)** Viability test of *Cymbidium* protoplasts treated with GA3 using FDA staining. **(C)** Relative expression levels of the *CsDELLA* gene induced by 10 μM GA3 were measured by qRT-PCR. **(D)** Relative expression levels of *CsFT*, *CsSOC1*, and *CsAP1* genes in response to GA3 induction by qRT-PCR. *Y*-axes indicate the relative expression levels; significant difference was assessed by Mann–Whitney U-test and indicated by asterisks; single asterisk (*) represents *p* ≤ 0.05, double asterisk (**) represents *p* ≤ 0.01; data are expressed as the mean of three biological replicates, with error bars indicating the SD values.

To test the feasibility of the *Cymbidium* protoplast system in cellular and molecular studies, functional identification of *CsDELLA* was conducted in *Cymbidium* protoplast. As DELLA-mediated GA signaling is known to affect flowering (Yamaguchi et al., 2014; Bao et al., 2020), we analyzed the role of *CsDELLA* in the regulation of GA to flowering-related genes. To determine the optimum concentration, the *Cymbidium* protoplasts were treated with different concentrations of GA3 (1, 10, and 100 μM), and the expression of *CsDELLA* in *Cymbidium* protoplasts was examined by qRT-PCR. *CsDELLA* expression was suppressed by the addition of GA3, and 10 μM GA3 was the best concentration for protoplast treatment (Figure 7A). To determine the reliability and repeatability, the viability of protoplasts post the treatment was estimated with FDA staining, and most protoplasts remained viable up to 24 hpt (Figure 7B).

Protoplasts treated with 10 μM GA3 and H_2_O control were collected at 6, 12, and 24 hpt, and the expressions of *CsDELLA* and flowering-related genes were analyzed by qRT-PCR. It was resulted that *CsDELLA* expression was significantly downregulated 3–6-fold from 12 to 24 hpt in the presence of 10 μM GA3 compared with controls at each time point (Figure 7C). However, the three key flowering-related genes *CsFT*, *CsSOC1*, and *CsAP1* were all significantly upregulated 1.3–15-fold from 12 to 24 hpt in response to 10 μM GA3 (Figure 7D). Whether GA promotes the expression of flowering related genes by suppressing the expression of *CsDELLA* remains unclear, and the role of *CsDELLA* in the regulation of GA to flowering-related genes needs further investigation. These results indicate the potential of our protoplast system for investigating cellular and molecular behaviors in response to phytohormones and other inducing factors.

### Efficient Transient Overexpression and RNA Interference for Gene Regulation Analysis in *Cymbidium* Protoplasts

**FIGURE 8:**
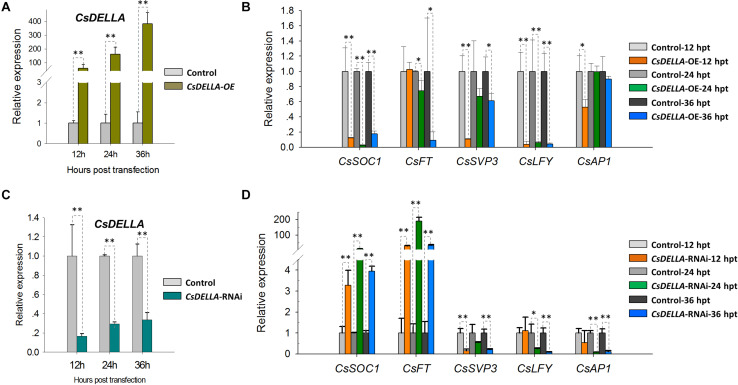
Regulation analysis of *CsDELLA* protein to the expression of flowering-related genes with qRT-PCR. **(A)** CsDELLA were transiently overexpressed in *Cymbidium* protoplasts and **(B)** the expression of *CsSOC1*, *CsFT*, *CsSVP3*, *CsLFY*, and *CsAP1* genes at 12, 24, and 36 hpt were examined with qRT-PCR. **(C)** *CsDELLA* expression was downregulated using RNAi and **(D)** the expression of *CsSOC1*, *CsFT*, *CsSVP3*, *CsLFY*, and *CsAP1* genes in the transfected protoplasts were examined with qRT-PCR. *Y*-axes indicate the relative expression levels; significant difference was assessed by Mann–Whitney U-test and indicated by asterisks; single asterisk (*) represents *p* ≤ 0.05, double asterisk (**) represents *p* ≤ 0.01. Data are expressed as the mean of three biological replicates, with error bars indicating the SD values.

To analyze the regulation of *CsDELLA* to flowering-related genes, *CsDELLA* was transiently over-expressed or knocked-down in *Cymbidium* protoplasts. The expression of flowering-related genes was examined with qRT-PCR. The recombinant plasmid pAN580-*CsDELLA* was transfected into *Cymbidium* protoplasts, and *CsDELLA* was successfully overexpressed 90–350-fold from 12 to 24 hpt (Figure 8A). The expressions of *CsSOC1* and *LEAFY* (*CsLFY*) were significantly downregulated 5–10-fold from 12 to 24 hpt, while those of *CsFT*, *CsSVP3*, and *CsAP1* were slightly downregulated. It indicated that the transient overexpression of *CsDELLA* suppressed the expression of *CsSOC1* and *CsLFY* (Figure 8B).

To identify the regulation relationships, *CsDELLA* was silenced with the RNA interference (RNAi) technology. The 243-bp *CsDELLA*-specific sequence was inserted into the RNAi vector pTCK303, and the vector pTCK303-*CsDELLA*-RNAi was transfected into *Cymbidium* protoplasts. It was resulted that the vector pTCK303-CsDELLA-RNAi (approximately 14.8 kb) was expressed in *Cymbidium* protoplasts, and *CsDELLA* was silenced to almost 70–80% (Figure 8C). The expression of *CsSOC1* was significantly upregulated 3–30-fold, and those of *CsFT* was greatly upregulated 50–160-fold from 12 to 24 hpt. However, *CsSVP*, *CsLFY*, and *CsAP1* were significantly downregulated (Figure 8D). These results indicated that silenced *CsDELLA* promoted the expression of *CsSOC1* and *CsFT*, *CsDELLA* probably positively regulates the expression of *CsSOC1* and *CsFT*. Our leaf base protoplast transfection system was confirmed to conveniently analyze gene expression regulation relationships, and to screen efficient vectors before genetic transformation.

## Discussion

Orchids are important ornamental and medicinal plants that are cultivated worldwide. Orchid biologists have placed great effort in protoplast, meristem, and tissue culturing for virus-free, hybrid, and rapid propagation of orchids (Lim et al., 1993; Shrestha et al., 2007; Yam and Arditti, 2009; Panattoni et al., 2013). With the release of *Phalaenopsis* (Cai et al., 2015), *Dendrobium* (Zhang et al., 2016a), and *Apostasia* (Zhang et al., 2017) whole genome sequences, highly efficient measures for rapid functional genomic studies are required. However, stable transgenes in orchids remain challenging because of their long-term growth cycles and difficulties in callus induction and plant regeneration (Kobayashi et al., 1993; Yam and Arditti, 2009). Accordingly, versatile protoplast systems have provoked attention. Herein, we performed a comprehensive parallel study to compare various tissues/organs of different orchid species as source materials for protoplast isolation and transfection.

Although appropriate optimizations have been determined for higher yield and viability of protoplasts isolated from different tissues of numerous species, the basic procedures for protoplast isolation have undergone little change since the first report (Nagata and Takebe, 1971; Kanai and Edwards, 1973; Sheen, 2001; Chen et al., 2006; Yoo et al., 2007). Source material is the most critical factor affecting protoplast release (Jeon et al., 2007; Pindel, 2007; Yoo et al., 2007; Zhang et al., 2016b). Various tissues of *in vitro* grown plantlets, including leaf tissues, flower pedicel, and column (Pindel, 2007), and *ex vitro* grown leaves and flower petals (Nanjareddy et al., 2016) as well as suspension cultured cells (Mazarei et al., 2008; Wang et al., 2015; Yao et al., 2016) have been used, and protoplasts have been isolated from various plant species (Table 1). In the present study, protoplasts were successfully isolated from various *ex vitro* tissues of orchids via the selection of suitable source materials and the optimization of enzymatic conditions. Using the improved method based on past reliable protocols, the tender leaf base gave higher yields (∼2.50 × 10^7^/g FW) compared to young leaves (∼3.22 × 10^6^/g FW), flower pedicels (∼5.26 × 10^6^/g FW), and root tips (∼7.66 × 10^5^/g FW) of *Cymbidium* orchids under optimal conditions (Figure 2). In addition to high yielding protoplasts isolated from *Cymbidium* flower petals (3.3 × 10^7^/g FW) (Ren et al., 2020), we successfully obtained sufficient tissue- and organ-specific protoplasts. Because orchids bloom only once a year and flowering lasts less than 1 month, the leaf base is the most suitable source material as it is available all year round for the isolation of high yielding viable orchid protoplasts. Moreover, leaf base protoplasts are of long-term health, which was mostly on non-differentiated stem cells. Since the ultimate goal of a protoplast isolation and transfection system is for plant regeneration, the totipotency of leaf base protoplast improved their probability of developing into mature plants.

The major factors affecting protoplast preparation are osmotic pressure, enzymatic concentration, and their combination in the enzyme solution, as well as the enzymolysis time (Kantharajah and Dodd, 1990; Davey et al., 2005). Herein, a concentration gradient of D-mannitol was used. Appropriate D-mannitol concentration can maintain the balance of interior and exterior osmotic pressures of protoplasts to prevent them from rupture or collapse (Huang et al., 2013). The optimum values for various tissues differed (Figure 2). Additionally, concentration gradients of cellulose-R10 combined with macerozyme-R10 were included. The increase in total enzyme concentrations resulted in increased protoplast yield and viability, whereas excess enzymes led to the phytotoxicity of enzymes on the membrane of protoplasts (Zhu et al., 1997; Raikar et al., 2008). The optimum concentrations of cellulose-R10 combined with macerozyme-R10 were determined to be 1.2% (w/v) and 0.6% (w/v), respectively (Table 1). Abundant calcium oxalate crystals accumulated in the ruptured protoplasts, which could puncture other protoplasts (Pindel, 2007; Lin et al., 2018b), and were observed in the protoplast solution (Supplementary Figure 1). Peroxides released by the ruptured protoplasts could also damage the protoplasts (de Marco and Roubelakis-Angelakis, 1996; Yasuda et al., 2007). To address these problems and protect protoplasts from rupturing, a suitable concentration of the reducing agent 2-mercaptoethanol was added to the enzyme solution. The protoplast yields markedly increased with the addition of 2-mercaptoethanol (Figure 3C). With this optimized protocol, high yielding viable protoplasts were obtained from the leaf base tissues of *Phalaenopsis* (1.83 × 10^7^/g FW) and *Paphiopedilum* (1.10 × 10^7^/g FW) (Figure 4) orchids, and other economically important monocot crops, including maize (3.25 × 10^7^/g FW) and rice (4.31 × 10^7^/g FW) (Figure 5). The isolation efficiency was higher or comparable to that of previous results (Table 1). This indicated the wide application of this protoplast isolation protocol for other orchids and monocot crops.

Protoplast-based transient expression system enables the molecular, cellular, and functional identification of genes/proteins in plants (Lin et al., 2018b). The emerging advantages of PTES have led to establishing a robust transient expression system using the leaf base protoplasts of *Cymbidium* orchids. Protoplast transfection efficiency greater than 50% is required for reliable and reproducible experimental data (Yoo et al., 2007). Hence, plasmid NDA expressing GFP was transfected into the protoplasts using a PEG-mediated method. Factors affecting PEG-mediated transfection, including incubation time, final PEG4000 concentration, and amount of plasmid DNA were optimized (Huang et al., 2013; Li et al., 2018; Page et al., 2019). A maximum transfection efficiency greater than 80% was obtained (Figure 6A), which is equivalent to or greater than that reported previously (Wu et al., 2017; Li et al., 2018; Lin et al., 2018b). Although most of the transfected protoplasts were almost translucent with few auto-fluorescent signals (Figure 3D), this PTES was successfully used for protein subcellular localization (Figure 6C). Moreover, we analyzed the induction of GA3 to the expression of *CsDELLA* and flowering-related genes in *Cymbidium* protoplasts (Figure 7). Moreover, we found that transient overexpression of *CsDELLA* significantly downregulated the expression of *CsSOC1*, *CsFT*, and other flowering-related genes (Figure 8A), whereas the silencing of *CsDELLA* resulted in the upregulated expression of *CsSOC1* and *CsFT* (Figure 8B). Thus, GA3 promotes the expression of flowering-related genes by suppressing *CsDELLA* expression (Supplementary Figure 4). Taken together, the leaf base protoplast isolation and transfection system described in the present study is robust, reliable, and sustainable for molecular and cellular characterization of genes/proteins.

## Data Availability Statement

The original contributions presented in the study are included in the article/Supplementary Material, further inquiries can be directed to the corresponding author/s.

## Author Contributions

RR and JG carried out all the experiments and drafted the manuscript. CL, YW, and JJ contributed to the data analyses. DY, KL, SA, GZ, and FY were involved in co-ordination and supervision of the work and review of the manuscript. All authors have read and agreed to the published version of the manuscript.

## Conflict of Interest

The authors declare that the research was conducted in the absence of any commercial or financial relationships that could be construed as a potential conflict of interest.

## Supplementary Material

Supplementary Figure 1 Schematic of recombined vectors. **(A)** Schematic of GFP control, endoplasmic reticulum marker AtWAK2-GFP, plasma membrane marker AtPIP2A-GFP. **(B)** Schematic of recombined vector expressing CsDELLA-GFP fusion protein for subcellular localization analysis, and CsDELLA protein for transient over-expression (OE). **(C)** Schematic of the pTCK303-CsDELLA-RNAi vector construction.

Supplementary Figure 2 Protoplasts isolated from the young leaves of *Cymbidium* orchids. Most of the protoplasts maintained integrity not only at **(A)** 3 h post releasing, but also at **(B)** 24 h post isolation. Arrows indicated the of calcium oxalate raphides.

Supplementary Figure 3 Cellular morphology comparison of protoplasts isolated from maize and rice. Bar, 20 μm.

Supplementary Figure 4 Conserved putative genetic regulatory networks of flowering in plants. Genes are represented by ovals. Lines with an arrow represent promotion of mRNA transcript, and those with a perpendicular bar represent repression. Red lines indicate regulation analyzed in this study.

Supplementary Table 1 Primer sequences used in this study.
