# Supplementary material for: Highly Efficient Leaf Base Protoplast Isolation and Transient Expression Systems for Orchids and Other Important Monocot Crops
Source: Front Plant Sci. 2021 Feb 15;12:626015. doi: 10.3389/fpls.2021.626015 (PMC7917215; doi:10.3389/fpls.2021.626015)
Supplement: Supplementary Figure 1 — Schematic of recombined vectors. (A) Schematic of GFP control, endoplasmic reticulum marker AtWAK2-GFP, plasma membrane marker AtPIP2A-GFP. (B) Schematic of recombined vector expressing CsDELLA-GFP fusion protein for subcellular localization analysis, and CsDELLA protein for transient over-expression (OE). (C) Schematic of the pTCK303-CsDELLA-RNAi vector construction. [file Data_Sheet_1.docx]

**Table S1** Primer sequences used in this study.

|  | **Primer name** | **Primer sequence（5’￫3’）*** |
| --- | --- | --- |
| **Subcellular localization** | PAN580-*CsDELLA*-F | CTTAAGTCCGGAGCTAGCTATGGGAAGTGGTGGGGACGGAA |
|  | PAN580-*CsDELLA*-R | TCGCCCTTGCTCACCATCAGTATCAACCCACCCAGGCCCAT |
| **Transient expression** | O-*CsDELLA*-PAN-F | CTCTAGAGACGTCTCGAGGATGGGAAGTGGTGGGGACGGAA |
|  | O-*CsDELLA*-PAN-R | TGTTTGAACTGCAGCCGGGTCACAGTATCAACCCACCCAGGCCCAT |
|  | O-*CsSOC1*-PAN-F | CTCTAGAGACGTCTCGAGGATGGTGAGGGGAAGGACGGAGATGA |
|  | O-*CsSOC1*-PAN-R | TGTTTGAACTGCAGCCGGGTCATCCTTTCATCAACTGTTGGG |
| **RNAi** | pTCK303-*CsDELLA*-RNAi-F | AACGATCGGGGAAATTCGAGCTCTCGTCGTCCTCATCCTCTTT |
|  | pTCK303-*CsDELLA*-RNAi-R | TGCAGGTCGACTCTAGAGGATCCACCAGCAACTTTTCGCATCG |
| **qRT-PCR** | Q-CsDELLA-F | CTCGTCGTCCTCATCCTCTTT |
|  | Q-CsDELLA-R | GGCGGTTTTGTAATTCTCTTG |
|  | Q-CsFT-F | GGCAGGAAGTGATGTGCTATG |
|  | Q-CsFT-R | GCGAAGTCCCTGGTGTTGA |
|  | Q-CsSVP-F | GCTTTCTATTCTTTGCGATGC |
|  | Q-CsSVP-R | GGTTTGGATATGTTCCTTGACTG |
|  | Q-CsSOC1-F | GGGAAGGACGGAGATGAGG |
|  | Q-CsSOC1-R | ATCGCAGAGCACGGACAAC |
|  | Q-CsAP1-F | CTGAGGTCGCTCTAATCGTG |
|  | Q-CsAP1-R | GTGCCGTTTGCTCTTCTGT |
|  | Q-CsLFY-F | TTGGGGATTTGGGGTTTAC |
|  | Q-CsLFY-R | GATGATTGTGATGGTGGGAG |
|  | CsUBQ-F | CCGGATCAGCAAAGGTTGA |
|  | CsUBQ-R | AAGATTTGCATCCCTCCCC |

* The sequences on the lines are the fusion sequence.

**
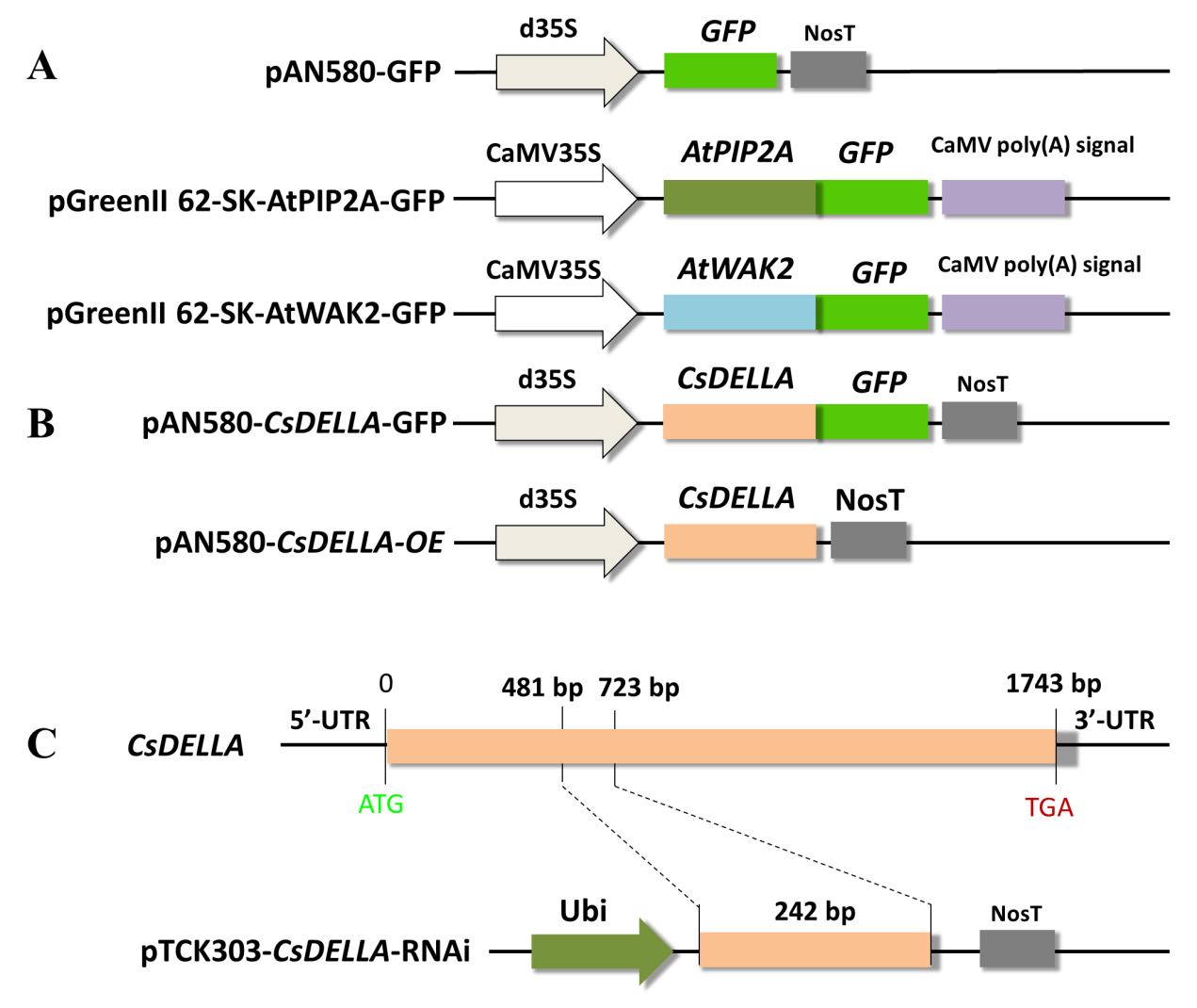
**

**Supplementary Figure 1** Schematic of recombined vectors. **(A)** Schematic of GFP control, endoplasmic reticulum marker AtWAK2-GFP, plasma membrane marker AtPIP2A-GFP. **(B)** Schematic of recombined vector expressing CsDELLA-GFP fusion protein for subcellular localization analysis, and CsDELLA protein for transient over-expression (OE). **(C)** Schematic of the pTCK303-CsDELLA-RNAi vector construction.

**
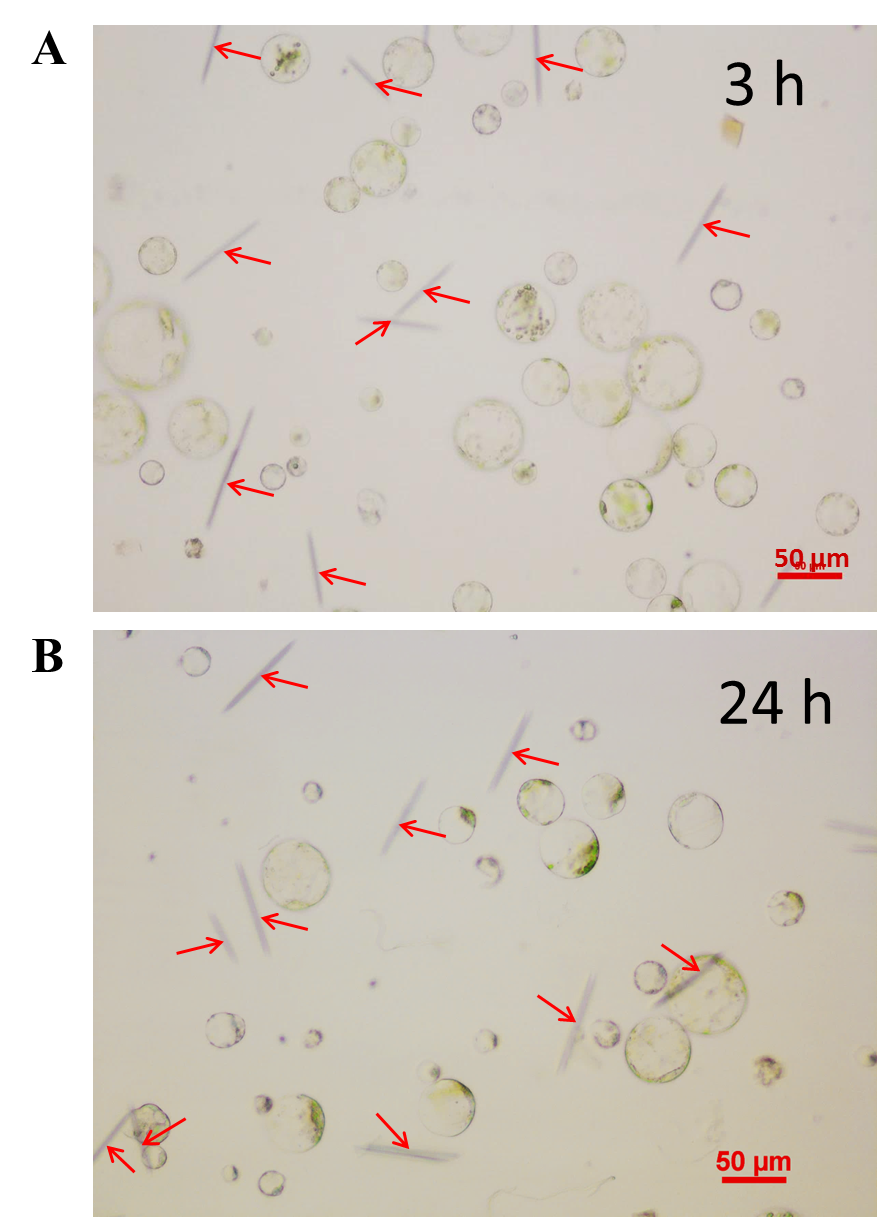
**

**Supplementary Figure 2**

Protoplasts isolated from the young leaves of *Cymbidium* orchids. Most of the protoplasts maintained integrity not only at (A) three hours post releasing, but also at (B) 24 hours post isolation. Arrows indicated the of calcium oxalate raphides.


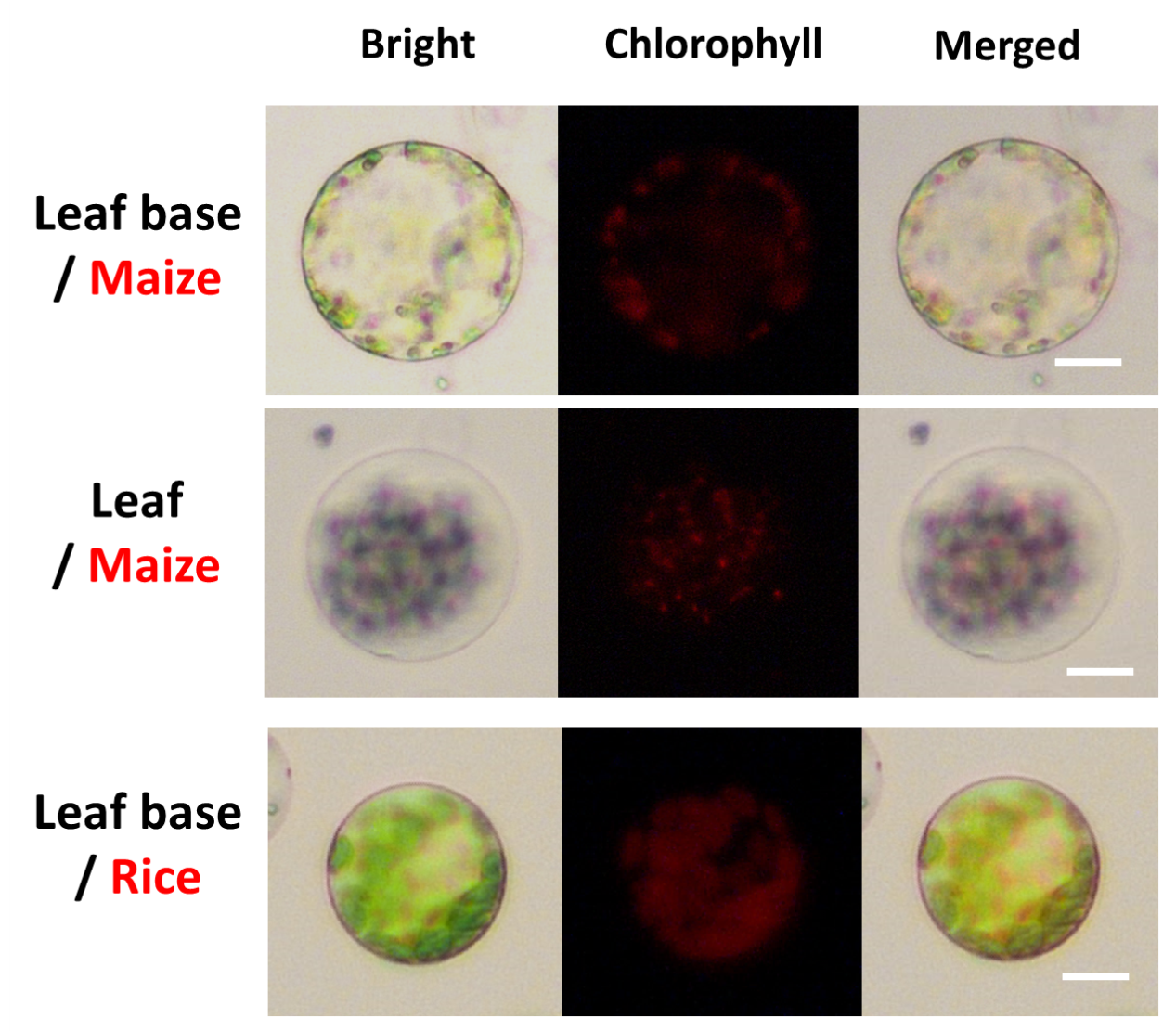


**Supplementary Figure 3** Cellular morphology comparison of protoplasts isolated from maize and rice. Bar = 20 μm.


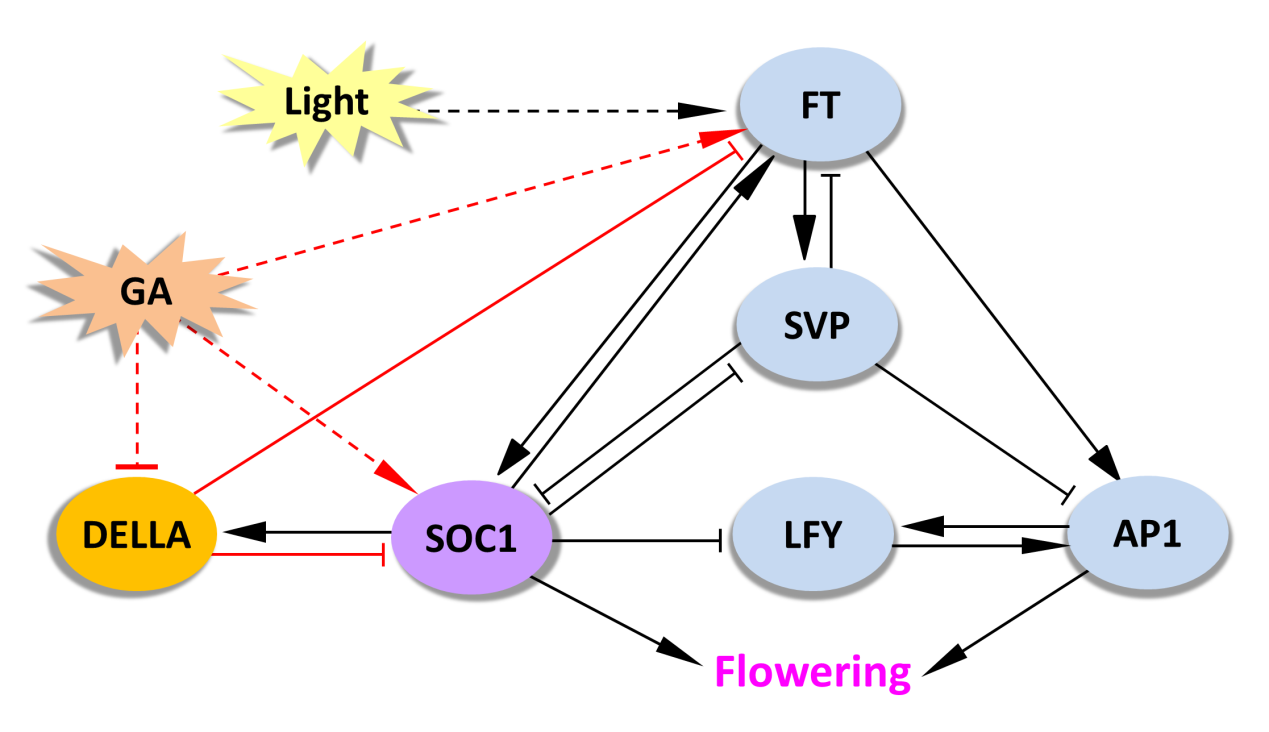


**Supplementary Figure 4** Conserved putative genetic regulatory networks of flowering in plants. Genes are represented by ovals. Lines with an arrow represent promotion of mRNA transcript, and those with a perpendicular bar represent repression. Red lines indicate regulation analyzed in this study.
